# Factors that influence the delivery of chiropractic services to equity-deserving groups in Canada: a qualitative study

**DOI:** 10.1186/s12998-025-00582-3

**Published:** 2025-05-15

**Authors:** Nora Bakaa, Stephanie DiPelino, Danielle Southerst, Silvano Mior, Lisa Carlesso, Joy MacDermid, Luciana Macedo

**Affiliations:** 1 Department of Rehabilitation Sciences, McMaster University, 1280 Main St. W, Hamilton, ON Canada; 2 Institute for Disability and Rehabilitation Research, Ontario Tech University, Oshawa, ON Canada; 3 Department of Research and Innovation, Canadian Memorial Chiropractic College, Toronto, ON Canada; 4 School of Physical Therapy, Western University, London, ON Canada

**Keywords:** Chiropractic, Qualitative research, Cultural competence, Equity, Diversity

## Abstract

**Background:**

Health inequities disproportionately impact equity-deserving groups, which include individuals marginalized due to race, ethnicity, Indigenous identity, sex and gender, socioeconomic status, and other social determinants of health. This qualitative study aimed to explore Canadian chiropractors’ experiences and perceptions in delivering care to equity-deserving groups and identify individual and institutional factors that may influence care delivery.

**Methods:**

We utilized interpretive description for data development, sampling, collection, and analysis. Participants were recruited as part of a larger mixed-methods research study, where we conducted a cross-sectional survey assessing Canadian chiropractors' diversity and cultural competency. We used maximum variation sampling to recruit chiropractors who indicated their interest in participating in the qualitative study.

**Results:**

Fourteen participants (N = 7, female) were included in this study, ranging from 28–64 years of age. We identified three major themes: 1) *Perceived role of institutions to advance cultural competency*, describing the approaches and strategies of professional associations and educational institutions in making changes concerning diversity, equity, and inclusion (DEI), 2) Fostering a culturally responsive clinical practice, describing factors that impact the delivery of care to equity-deserving groups (e.g. ensuring clinicians’ cultural awareness and sensitivity, promoting culturally competent behaviours, and understanding patients’ cultural values), and 3) Understanding the contextual determinants in accessing care (e.g., socioeconomic status, lack of accessibility, patient advocacy).

**Conclusion:**

The results suggest that culturally congruent care involves top-down and bottom-up approaches that integrate DEI practices at institutional and clinician levels. Specifically, the incorporation of DEI training within curricula, the development of policies that foster diversity, the engagement of equity-deserving groups to understand unique cultural needs, and tailoring treatments to each patient rather than a one-size-fits-all approach.

## Introduction

Canada’s growing population demands an accessible healthcare system that provides equitable and culturally sensitive healthcare services for all Canadians. Health inequities disproportionately impact equity-deserving groups, which include individuals marginalized due to race, ethnicity, Indigenous identity, sex and gender, socioeconomic status, and other social determinants of health [1–7]. While equity-seeking groups actively advocate for systemic change to address barriers to healthcare access, equity-deserving groups inherently warrant equitable care based on social justice and inclusion principles [8]. These terms are often used interchangeably, but their distinction is crucial: equity-seeking reflects activism for change, whereas equity-deserving emphasizes the fundamental right to healthcare without requiring advocacy. In this study, we used the term "minority community," defined as: differences among groups of people and individuals based on ethnicity, race, socioeconomic status, gender, exceptionalities, language, religion, sexual orientation, and geographical area [8]. This terminology reflects the perspectives shared by participants while recognizing the evolving discourse around equity-deserving and equity-seeking populations.

Equity-deserving groups have a greater prevalence of chronic conditions [9], report higher levels of pain and disability and experience more significant barriers to accessing rehabilitation services [1–7]. These health inequities can be the result of systemic discrimination or social injustice [10, 11], compounded by language barriers, accessibility [12], clinician bias [13–16], cultural differences [12], and lower health literacy [17]. Addressing these disparities requires integrating diversity, equity, and inclusion (DEI) principles into healthcare frameworks to promote culturally responsive care. A commonly used conceptual framework for cultural competency, developed by Schim et al. [18–20], highlights four key constructs that shape cultural competence: (1) cultural diversity experience, (2) cultural awareness, (3) cultural sensitivity, and (4) culturally competent behaviours. Cultural diversity experience represents a clinician’s personal background and exposure to equity-deserving groups, which can shape their cultural competency. Cultural awareness refers to a clinician’s understanding of cultural differences, while cultural sensitivity reflects their attitudes toward respecting those differences during clinical interactions. Lastly, culturally competent behaviours reflect the application of these principles in clinical practice, ensuring care is adapted to meet patients' cultural needs. By incorporating these elements into chiropractic education and practice, providers can foster an inclusive and equitable healthcare environment that effectively meets the needs of equity-deserving groups.

Our previous work highlights a significant lack of diversity in the Canadian chiropractic profession, with Caucasians comprising 80% of the workforce and notable underrepresentation among Filipino, Latin American, Southeast Asian, Arab, Chinese, South Asian, Indigenous, and Black chiropractors [16]. Women remain underrepresented (45%), with only 6% identifying as gender minorities, including trans men, trans women, non-binary, gender fluid, Two-Spirit, and other cultural gender identities. Beyond demographic gaps, most chiropractors (72–78%) reported health disparities in care-related outcomes, citing cost and language barriers as major obstacles to equitable care [15]. While cultural awareness and sensitivity were moderately high, culturally competent behaviors in practice were lower, particularly among men, more experienced clinicians, and Caucasians, while prior DEI training was associated with higher scores [15]. These findings highlight the urgent need for enhanced DEI strategies to improve culturally responsive care.

Although policies related to DEI have evolved in recent years, there is a need to enhance educational content within entry-level rehabilitation programs to address health inequities effectively [21, 22]. Current research describes how ableism [23], ageism [24, 25], race and ethnicity [26], and perceived discrimination against equity-deserving groups can lead to a reduction in healthcare-seeking behaviours or acceptance of limited treatment options presented by providers. While training in DEI and fostering diversity can enhance cultural competency within a profession, there remains a lack of understanding of the perceptions and experiences of Canadian chiropractors in providing care to equity-deserving groups. Therefore, this study qualitatively examines Canadian chiropractors’ experiences and perceptions in delivering care to equity-deserving groups and identifies individual and institutional factors that may influence care delivery.

## Methods

### Study design

This study adhered to the Standards for Reporting Qualitative Research (SRQR) guidelines [27] and employed interpretive description [28–30] as its qualitative research approach, with a constructivist lens. This approach facilitates the exploration of chiropractors’ experiences in the delivery of rehabilitation to equity-deserving groups in Canada, yielding insights for addressing health disparities.

### Ethical considerations

Ethics approval was obtained from the Hamilton Integrated Research Ethics Board (#13,042).

### Recruitment/sampling

A convenience sample of chiropractors was recruited as part of a cross-sectional survey assessing health disparities among Canadian chiropractors [15, 16]. The survey was sent to members of the Canadian Chiropractic Association (n = 3143, 41% response rate). Those who completed the study were asked to provide their email if they were interested in participating in a qualitative interview (n = 282). Between November 2021 and April 2022, the research team first stratified the 282 interested participants by sex and age to ensure maximum variation. From this stratified pool, a random sample of 155 individuals was contacted (one email), and 14 participants were ultimately included in the study.

### Data collection

Data were collected using in-depth semi-structured interviews (45 min—1 h). The interview guide was developed based on previous literature that assessed cultural competency within rehabilitation [12, 31] and from the authors’ prior understanding and experience (see Appendix A). For example, participants were asked questions such as: “How do you approach the delivery of your health care services to patients from different cultural groups?” and “Do you think you have sufficient cultural knowledge to provide treatment?” Questions were designed to capture clinicians' experiences, perceived barriers to equitable care, and strategies for fostering cultural competence. The interviews included open-ended questions that encouraged participants to reflect on their experiences providing care to minority groups, their challenges, and how cultural factors influenced clinical interactions. Additional questions examined barriers to access, clinician training, and the role of institutions in supporting DEI initiatives. Participants were provided with the interview guide before the interview to ensure rich reflection, development of detailed responses, and sharing examples. Participants also completed demographic information (e.g., age, sex, gender, practice community, etc.) to allow for a description of participants. Data was collected until saturation, meaning no new codes or themes were identified as determined by NB, SD, and DS.

### Researcher characteristics

NB is a practicing chiropractor with six years of clinical experience and a PhD in Rehabilitation Sciences. She identifies as a female/woman and a Canadian refugee. SD identifies as a female/woman and is a research coordinator with a master’s degree in public health (https://chiropractic.ca/dr-nora-bakaa/). DS identifies as female/women and is a chiropractor with a master’s degree in public health. All researchers who conducted the interviews and/or primary analysis have formal training or experience in qualitative research methodology.

### Data analysis

All interviews were audio-recorded and transcribed verbatim using Zoom [32], then reviewed for accuracy by an independent paid reviewer who was not a member of the research team. Transcripts were imported into the qualitative software Dedoose (https://www.dedoose.com) to manage, store, and code the data. Inductive thematic and constant comparative analysis were used to analyze the data and report study findings [30]. Coding was informed by the research question, the cultural competency framework [19], the interview guide, prior knowledge of the researchers, and the transcripts. Initial coding was conducted inductively, with codes derived from the transcripts through line-by-line analysis. In later phases, constructs from the cultural competency framework were integrated to refine themes and subthemes and guide interpretation of findings.

Themes were developed using Braun and Clarke’s (2006) six-phase method for thematic analysis [33]. In line with interpretive descriptive methodology, our analysis described participants’ experiences and perceptions in delivering care to equity-deserving groups and the key factors influencing culturally competent care delivery, acknowledging that meaning is co-constructed in the research process. The first step involved familiarization with the data, where NB, DS, and SD conducted an in-depth, line-by-line reading of the transcripts to ensure immersion in participants' narratives. This was followed by generating initial codes, in which meaningful text segments were assigned conceptual labels reflecting key ideas. Initial codes were developed based on the interviews, the cultural competency framework, and the researchers’ (NB, DS, and SD) understanding of this topic. Next, searching for themes involved grouping similar codes into broader categories that captured shared patterns across the data. These categories were then refined in the reviewing themes phase, where the research team examined their coherence, ensuring they were well-supported by the data. In the defining and naming themes phase, themes were further refined to enhance clarity and interpretability. Finally, in producing the final themes, findings were reviewed by LM, SM, and LC, who provided feedback on the appropriateness of selected quotes and thematic interpretations. The entire research team engaged in a final review and revision process to ensure that themes were conceptually sound, rigorously developed, and accurately represented participants’ experiences.

### Rigour and trustworthiness

To ensure rigour and trustworthiness [30], we aligned findings with interpretive description assumptions and prior research on cultural competence, ensuring themes reflected the participant’s narratives, established literature, and the researchers’ knowledge. Sampling and design were aligned through purposeful sampling with maximum variation, ensuring diverse perspectives were captured. Member checking involved sharing the themes with participants to confirm accuracy and resonance with their experiences, and participants provided no additional feedback. An audit trail was maintained to track decisions throughout the data collection and analysis phases. Reflexive journals were maintained to acknowledge and bracket researcher (NB, SD, and DS) assumptions and positionality, thereby minimizing interpretive bias and enhancing analytic rigour. Additionally, coding was done independently by two researchers (NB, SD, or DS) to enhance the dependability of the results. Lastly, the use of sufficient participant quotes ensured that they remained directly connected to the data (Table 1).

**Table 1:** Sample of summary of quotes for each identified theme

Theme	Subtheme	Sample Quote
Recognizing the need for enhanced institutional cultural competency	Navigating systemic racism among Indigenous communities	**P007:** Encourage Indigenous kids to go into the health field because we need to break down that systemic racism and those issues that are happening in our healthcare system **P007:**Reinstate the coverage for Indigenous people under their current insurance provider is not insured health benefits so it's NIHB be that's a program delivered through health Canada. And they used to have coverage for chiropractic for Indigenous people that was probably up until the 90 s, at some point, and then in the 90 s, with all the cuts, with the Conservative Government, chiropractic was not a surprise that it was slashed **P002:** No, I do not think chiropractic as a profession has figured this out yet. It's predominantly a white profession. It predominantly treats white people
	The need for advocacy to reduce systemic barriers	**P013:** Well, certainly a better funding for a chiropractor, of course, would be a better issue or improved insurance benefits to relieve financial barriers, for those who have to have insurance **P007:** So, for example, the [Canadian Chiropractic Association] has a mandate that chiropractors, will be on everybody's healthcare team, by the year 2023, when most of the people that I’ve spoken to up here in northern Manitoba never even heard of a chiropractor, that's not going to happen by 2023 if we don't start moving towards building education in these communities to show how we can help, how we can work with them, how we can help them, how we can better their health care services ***P014:*** I think, this is a call on the advocacy groups, this is a call on the national regulatory bodies now to say listen, we need to help you I can give you the skills to give to your members, in order for them to help identify what biases that they have **P002:** I would then ensure that we have a policy on you know, diversity, equity and inclusion I would, I would ensure that as a clinic were going and doing outreach to some of these communities and yeah. Just being more involved right, more hands on in the community and providing services and support
	Strengthening DEI competencies in professional training	**P03:** Yeah if someone, for some people pain is part of life, and then they just seek relief to be able to achieve their daily activities. And that's kind of easier because their expectations are more realistic. Some other people if for them pain is something that should be avoided, and that is not normal and that they shouldn't not feel, then usually for them, it means that they need a lot more education and explanation about what's going on things like that, so they can have, they can bring down their expectation sometime people expect like one or two visits they'll get pain free but they've been in pain for months and so those people need to be reassured that pain is normal and you know to an extent, like it's normal to feel pain that it takes time to get better things like that. Much more education for that group of people that want to avoid pain at all costs that don't think it's normal **P002:** Just to create this level of awareness on racism and systemic discrimination and culture, and all of these things. But we have a long way to go, I think it needs to infiltrate our curriculum, more needs to infiltrate, so that you know people are trained on it right from the beginning, so when they graduate they're like oh yeah I'm good with this, I already know this right
	Advancing equity though diverse representation	**P013:** Chiropractic is predominantly a Western health care system and the chiropractic colleges, are going to be North America, Australia, many in Europe now that are serving Western European languages. I suppose that as people from cultural groups are coming to Canada, many, there are some who will choose to go into chiropractic and that will eventually in a generation or so increase the number of ethnically or ethnically minority-oriented practitioners. So, it's going to be a while, for that to catch up **P012:** And then also just internal privileges that some people are afforded to them. I had to have both my parents cosign for me and so their entire credit rating was looked at right, so if your parents aren't in a position to be you know co-signing and I guess the jig is up right. So, and there really wasn't any financial aid to speak of that I was aware of at least when I was going through, so I would imagine that that creates that big divide **P002:** We do not have good representation, even forget color, women just women in leadership is low, so you've got you know more than 50% of students in chiropractic colleges are female, yet they have no female role models and leadership, so what would encourage them to become leaders in the profession, if they don't see anybody that looks like them at the top. So, I think it's really important that these young people are able to visualize what they could be ***P011:*** You're creating a culture where those people are welcome isn't even the right word. Sought out right because right now it's like, oh, anyone can go into healthcare, and it's like, but can they like, think about the barriers that they have to pursuing higher education, the trauma they may have experienced, the low income they have to work their way through school. Whereas you know us rich white kids just gets paid, tuition gets paid by our parents, and we just get to the school and get straight A's because we're sleeping at night, you know what I mean
Fostering a culturally responsive clinical practice	Increased cultural awareness and sensitivity	**P011:** “But to patients who are from [Non-Western] cultures and, particularly, who have been marginalized and who have been traumatized in the colonized system that we have, those are greater indicators of positive outcomes than anything that comes out of your mouth or anything you teach them to do, because if they don't trust you, they won't actually tell you what's going on and they won't believe anything you say, and they will be afraid because without that trauma-informed lens on you know you don't know if they've had a bad experience with a chiropractor or a doctor.” **P005:** I think it's a lack of perspective, I think, maybe you know the rigors of becoming a doctor limit some people's exposure to the world and maybe they don't have experiences that are broad enough to to really understand that you know what they're learning in books may not be true. And you know, depending on what they're reading and what they believe they can have some very damaging ideas ***P008:*** I don't know if we ever have enough cultural knowledge, but I feel comfortable with treating anybody and everybody. I'm always learning. And so I guess I’m just trying to be sensitive and I, you know I feel like I haven't had any alarm bells go off with how I’ve dealt with things, but I feel like I’m always I guess yeah sensitive to me not knowing the right thing to do or say, but at least giving that the safe space to be able to manage it in a you know, whatever humane way so
	Developing culturally competent behaviours	**P011:** We need a culture that says, we get that not everyone has the same playing field and we're going to make it easier for the people for whom it is intrinsically harder, because that is the only way to make it equitable. Equitable doesn't mean the same for everybody, it means that everybody gets what they need, and once we have a culture and a desire and people actually actively deserving out, different perspectives and lived experiences, I think you will see more practitioners, because people will… Barriers will be removed and people will feel included, and it won’t be such an uphill battle and you know people from minority groups need healthcare practitioners from those minority groups, that is the fastest way to build trust and the fastest way to have better health outcomes for patients, when they trust their practitioner. So I mean it's a culture and a system shift and it starts with white wealthy healthcare practitioners getting it through their heads that they're not god's gift to mankind and taking some taking some education and just listening and humbling themselves and being open to the reality that they are privilege **P014:** We have portions of our intake form for languages in case they need [other] languages. If they need a form translated, a huge population over here is the Punjabi population, so we do have intake forms that are translated into that right. So, we try our best and in terms of accommodating for language barriers, but also for people that just need additional time for their appointments. […] We create larger appointment times for people with language barriers or people that seem to have more complex cases, that way they're heard to the same extent that a [native] English speaker is heard
	Understanding patients’ cultural values	**P006:** A mainstream North American perspective on health and how you reach those health outcomes, that's not necessarily the same approach or perspective that other people from other cultures and ethnicities view their health **P007:** In Indigenous health we practice what's called the medicine wheel, and that includes mind body spirit and emotion and so Western medicine is just getting into mind body medicine. We take that further we take that twice as far we go mind body spirit and emotion and then make sure, that ensures that all aspects of locations well-being are monitored, not just their physical body, but their mental health, their nutrition, and their and their emotions and their responses and we all know that stress pay plays a big role and so by understanding my teaching in indigenous medicines and of the medicine wheel that allows me to apply those principles to my patients and I’ve done this over the years **P003:** Pain is not always seen the same for some people part pain is just part of life it's something that is normal, and when it gets too much, when it affects their daily activity they will consult only then. And for some other culture, it seems like pain is something that should be avoided at all costs, like any pain is an issue
Contextual determinants of cultural competence in accessing care	Patients’ socioeconomic status	**P011:** I find even booking the booking process can sometimes be a challenge for people if they aren't you know middle, upper middle class, you know phones with Internet, very tech savvy. For them online booking is great, but for older generations are people who don't have a lot of technology it's a real problem. And, and the same with follow up in the same, like exercises so again technology […] [Y]ou can send exercises, but again, you need a computer, you need to set up a password, you need to create an account, you need to login, you need to have access **P014:** The people working a lot in the area over here just to make ends meet and as a result of that the attendance is poor but also, they don't get better because they have the constant bombardment of what's… The stimuli that's also causing detriment to their house, for example, if they have a working job that's repetitive in nature and their injuries or injury that's a result of a job that's repetitive in nature there in the cyclical pattern of never getting better, even if you provide them with the full course of treatment, for example. You give them the skills and the tools that they need to be successful, to alleviate pain, however, they can’t alleviate that pain, because their bodies are just not designed to work 16 h a day with repetitive motion
	Lack of accessibility limiting access to care	**P014:** So, in terms of equitable patient care, we do have it for physical disabilities type of thing, where we have. Individuals that that we have we make it we have bigger rooms there, so we have smaller rooms, we have bigger and bigger rooms are for those individuals that need that require them more space. we have chairs that are a little bigger in size for obese patients, we have you know assistant devices here that will help individuals that find that they have a little bit of a functional impairment; to get in so we do make provisions for individuals that have that are a little bit of need, in certain ways mentally speaking and cognitive cognitively speaking. We're dealing with stroke victims, we're dealing with patients that may have certain impairments, such as reading or writing etc. We accommodate that by asking right I’m trying to adapt the needs; I can't tell you what the needs are because I don't know until I ask **P007:** So having the profession so far away from the [Indigenous] communities is what would be a major barrier **P009:** If there's a language barrier then usually I ask if they have somebody there okay to come in to help translate, especially with the beginning stages and then, once we're more comfortable and I go maybe try to learn some phrases in their language to help say too much or pain or things like that they get through
	Chiropractors as advocates for patient equity	**P004:** If they have other health issues outside of the chiropractic practice if they don't have a medical doctor, that's another big problem, the lack of medical doctors, you know, I do try to find them or guide them towards resources that may help them

## Results

Fourteen participants were recruited and participated, with a mean age (range) of 42.2 (28–64) years. Among those, seven identified as female/woman (50%), with an average of 16.5 (SD 9.5) years in clinical practice. Six participants identified as Canadian and European, one as South Asian, one as Canadian and African, one as Southeast Asian, one as mixed Caribbean, one as Indigenous, and two as Canadian. Participants were located across Canada (Ontario (n = 5), Nova Scotia (n = 2), British Colombia (n = 2), New Brunswick (n = 1), Manitoba (n = 1), Nunavut (n = 1), Alberta (n = 1), no response (n = 1)). One participant identified as having a disability, and one preferred not to respond. Participants reported working in various clinical settings ranging from interdisciplinary rehabilitation to interdisciplinary complementary and alternative medicine clinics, with three participants working as solo practitioners. Aggregate data was provided for demographic categories to avoid identifying participants.

Three overarching themes were identified: 1) Perceived role of institutions to advance cultural competency, 2) Fostering a Culturally Responsive Clinical Practice, and 3).

**Fig. 1:**
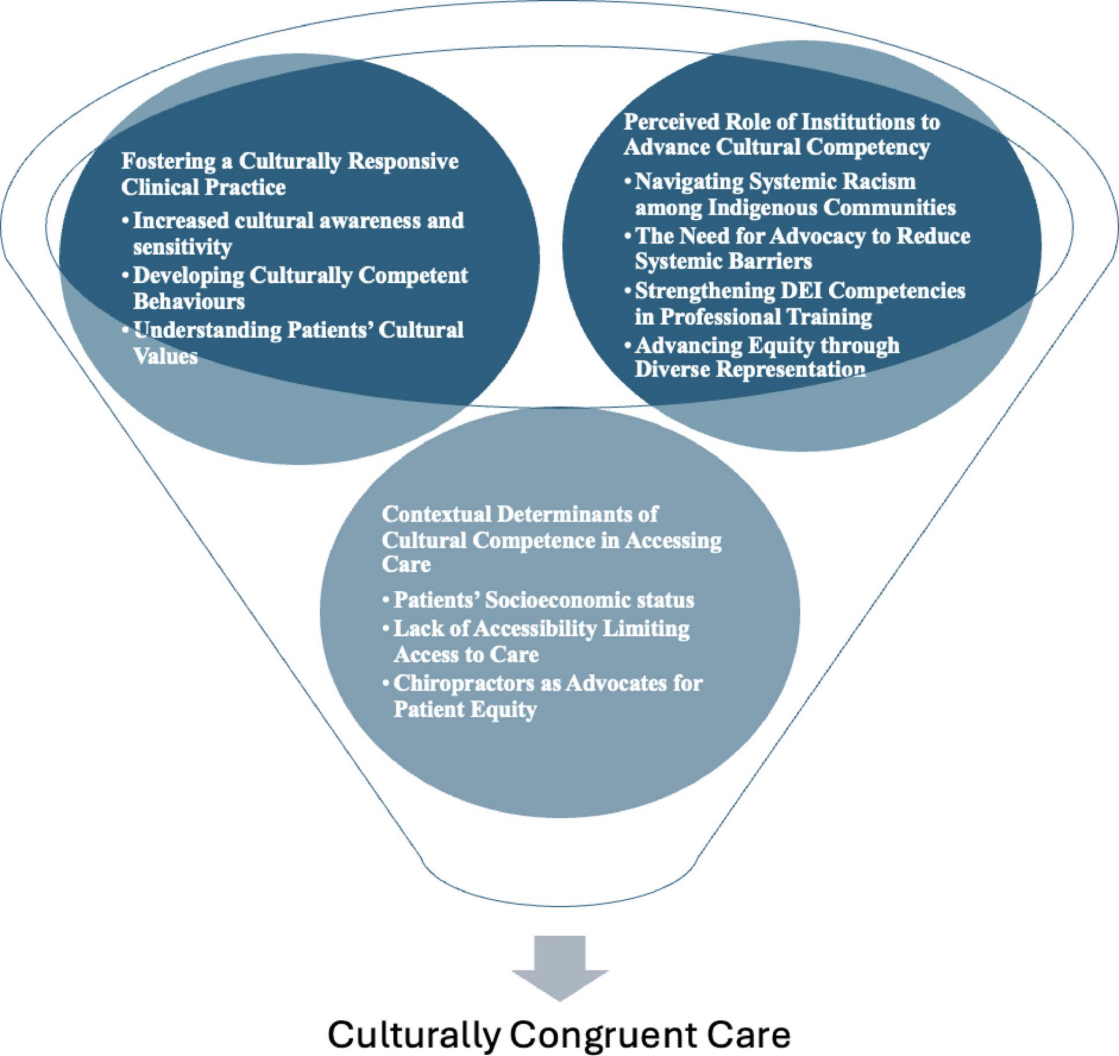
The themes associated with potentially improving the provision of culturally competent care among Canadian chiropractors

Contextual Determinants of Cultural Competence in Accessing Care (Fig. 1). These themes and related subthemes potentially facilitate the provision of culturally congruent care.

### Perceived role of institutions to advance cultural competency

The theme, *Perceived role of institutions to advance cultural competency*, captures participants’ views of an institution’s ability to understand, respect, and engage with individuals from diverse cultural backgrounds and implement policies to foster an inclusive environment. Institutions included chiropractic associations, regulatory bodies, and educational organizations. Participants described how structural barriers within these institutions, including limited diversity in leadership and among clinicians, inadequate DEI policies, and systemic exclusion of equity-deserving voices, can directly or indirectly affect clinicians’ ability to deliver equitable care. We identified four subthemes: 1) Navigating Systemic Racism among Indigenous Communities, 2) The Need for Advocacy to Reduce Systemic Barriers, 3) Strengthening DEI Competencies in Professional Training, and 4) Advancing Equity through Diverse Representation.

### Navigating Systemic Racism among Indigenous Communities

Systemic racism is a deeply rooted, structural form of discrimination and inequality that is woven throughout our societal institutions, policies, practices, and norms [34]. Participants highlighted the impact of systemic racism on Indigenous communities, particularly their mistrust of the Canadian healthcare system. One participant emphasized how obtaining informed consent from Indigenous patients might be misconstrued due to their past trauma affecting their receptivity to information*:*

***P011*:***“With First Nations patients, just because you give a report of findings, benefits, risks, and alternatives and go through a consent form and they sign it, does not mean you have informed consent. You do not have informed consent a lot of the time. Because they're in fight or flight, and they're just trying to get through that visit and get out, they're not hearing you, and they're just going to go with whatever you say.”*

These past traumatic experiences, compounded over years of oppression, shape how Indigenous communities perceive patient-clinician interactions.

### The need for advocacy to reduce systemic barriers

Participants emphasized the need for chiropractors to advocate for systemic barriers and health inequities. They highlighted the importance of advocating for change in government policies and publicly acknowledging biases in the delivery of chiropractic care, systemic racism, and engaging with equity-deserving groups.

One participant stressed the need to advocate for better access, citing frustration with bureaucratic challenges and misallocating funds, hindering patient care:

***P011:**** “I want to lay a fairly significant chunk of the responsibility at the foot of the government. […] I really didn't see any improvement in patient access to the care they need [with a change in government parties] when the care they need is not funded through the provincial system. And it inordinately affects people of minorities, with less means and less accessibility and less privilege.”*

Another perspective emphasized the importance of engaging equity-deserving groups to identify current healthcare needs.

***P002:**** “I think we should hear the patient's voices more. You know, let's get some patient groups to talk to us and to share these types of stories and to have more contact with these diverse communities. […] Rather than us going in and saying you need chiropractic, you know, to me that's a selfish and self-serving way of going about it.”*

This participant described critical allyship, emphasizing that engaging equity-deserving populations in advocacy efforts can help dismantle systemic inequities and challenge biases. Participants suggest engaging with equity-deserving groups to identify their needs and then advocating to key decision-makers to improve health inequities.

### Strengthening DEI competencies in professional training

Participants discussed educational strategies that chiropractic institutions could implement to address DEI, sharing undergraduate and continuing education insights. These educational opportunities were considered key to initiating dialogue and raising awareness of biases, with a suggestion that such training should be mandatory before graduation:

***P014:**** “The problem with a lot of these DEI trainings is that it's not mandatory. Where I think that these things should be pushed into mandatory. I don't think it's only for you know BIPOC [Black, Indigenous, People of colour] or marginalized people or immigrant populations, I think it's for everybody, we need training on the LGBTQ2*+ *[Lesbian, Gay, Bisexual, Transgender, Queer, Two-Spirit,* + *additional identities] community, we need training on a lot of the other populations out there that are marginalized because biases we all feel it the same way, just in different forms.”*

Other participants acknowledged the absence of DEI training opportunities during their undergraduate training and after graduating: ***P002:*** “*We have a long way to go; I think [DEI] needs to infiltrate our curriculum so that people are trained on it right from the beginning.”* While existing efforts to advance DEI within chiropractic institutions were acknowledged, concerns were raised regarding the adequacy of these interventions in addressing health inequity and the need for more training.

### Advancing Equity Through Diverse Representation

Increased cultural and gender representation at all levels of the chiropractic profession (e.g., among clinicians and within leadership roles) was considered necessary for a cultural shift prioritizing DEI. Participants described how representation influences perceptions of belonging within the profession and may impact clinicians’ ability to deliver care that aligns with patients’ cultural identities:

***P014:**** “I think [increased representation] needs to happen, and [at] all three levels. It needs to happen on a practitioner level, on an educational institutional level, and on these regulatory bodies as well. […] A lot of time, especially for my Caucasian counterparts here, some of them don't acknowledge that this is the thing right, [they think] that white privilege is not a thing...”*

Some participants believed that creating an environment where individuals from equity-deserving groups are sought after and valued, rather than merely welcomed, may help to dismantle systemic barriers. In turn, it may help to increase diversity within the profession:

***P011:**** “We have no DEI policy, and that is completely inappropriate. And it's one thing to say, oh anyone's welcome at the table, we're not going to slam the door on you. And it's another thing to really seek people out and say when there's a call for nominations, or when we're asking the government for public members to say we want lived experiences. We want diversity, like we value those qualities and that diversity perspective so much that when you're looking for people, please highly regard their lived experiences as much or more than their professional experiences.”*

Participants also highlighted the importance of having increased diversity among clinicians as patients often seek out practitioners like them in race, ethnicity or gender:

***P002:**** “It's been shown that you are more likely to seek out a health care provider that looks like you right, that has the same culture, or cultural background because you feel comfortable with them, you feel like they you know you can relate to them, and they can relate to you. And if the majority of chiropractors out there are White, there is a barrier.”*

Participants suggested that chiropractic institutions advocate for change by developing scholarships, improving education access, and outreach to equity-deserving groups. They recommended early engagement with equity-deserving groups to encourage aspiring students to consider chiropractic, with underrepresentation being linked to systemic barriers limiting access to higher education.

## Fostering a culturally responsive clinical practice

This theme of Fostering a Culturally Responsive Clinical Practice captures the practitioner-level strategies/factors that may influence the delivery of chiropractic care to equity-deserving groups. Three subthemes were identified under this theme: 1) Increased Cultural Awareness and Sensitivity, 2) Developing Culturally Competent Behaviours, and 3) Understanding Patients’ Cultural Values.

### Increased cultural awareness and sensitivity

Participants emphasized the importance of cultural awareness and sensitivity in their clinical interactions, mainly as it related to understanding the diverse backgrounds of their patients. This subtheme is structured around two key perspectives: how chiropractors develop cultural awareness and sensitivity and the challenges in achieving cultural sensitivity.

To develop cultural awareness and sensitivity, all participants highlighted the importance of comprehensive patient discussions extending beyond immediate complaints for delivering adequate care:

***P001:**** “I think it's important to raise awareness that, like as a clinician, yes, we’re focused on the patients and the human body coming in, but there are so many more factors that play into it and when we learn, you know, to take histories or do anything like that like we go through the checkboxes of like, what's your health history in your family? But maybe we need to start asking questions more about, like, what's your view of wellness or health?”*

Participants noted that their in-depth interactions during the clinical encounter empowered them to grasp culturally specific patient needs and tailor their approach to clinical management. Building trust was identified as a key component in building rapport that encourages patients to discuss various aspects of their health, contributing to more informed treatment, allowing patients to feel comfortable discussing various aspects of their health and contributing to more informed treatment decisions. However, participants also shared instances where a lack of cultural awareness and sensitivity led to harmful patient experiences, ultimately eroding trust in the profession and discouraging future care-seeking. One participant recounted an incident where a clinician’s failure to recognize the significance of religious attire resulted in a patient avoiding chiropractic care for years:

***P002****: “I had a patient who saw a chiropractor, and she wore a hijab, and the chiropractor basically said, well, I have to examine your neck and just proceeded to take off the hijab, and that is like absolutely the biggest, no, no, you know, it's like pulling the robes off a nun. Like you just don't right, and even though she said she told him that, you know, I can't remove the scarf, he said, well, there must be a provision because I'm a doctor and just proceeded to remove her scarf. And she literally ran out of the office and never saw a chiropractor again until a friend told her, there's a Muslim chiropractor that I've started seeing. So, she suffered from migraine headaches for seven years because of that experience, so you know, there's a great example of someone who just wasn't aware and didn't think it was a big deal to remove a headscarf.”*

Despite efforts to enhance cultural sensitivity, several participants acknowledged persistent clinician bias and limitations in self-assessing cultural competency. Some clinicians expressed uncertainty about cultural differences among equity-deserving groups, particularly in bridging cultural gaps in communication and treatment approaches. Language barriers were noted as a challenge to cultural sensitivity, though opinions varied. One participant (P013) expressed concerns about language barriers, suggesting that increased diversity discourages newcomers from learning the language of their host community***.*** This participant seems to overlook an individual's unique language acquisition and integration challenges, suggesting a limited understanding of the nuances involved in cultural adaptation.

### Developing culturally competent behaviours

Participants described the importance of culturally competent behaviours, but the definitions varied. For example, two opposing subthemes emerged when delving into the strategies they used to accommodate patients' cultural needs. One approach emphasized a patient-centered model, advocating for individualized care and communication that aligns with patients' unique experiences and needs.:

***P010:**** “I have to admit, I sort of struggle with the idea [of equitable care] […] I guess the procedures are sort of in place to try and make things easy for everybody, but then that obviously brings up the fact that there are some people who have different needs, and so providing the same thing for everyone doesn’t necessarily produce quality care.”*

The other approach reflected a uniform tactic, where cultural factors like language and beliefs were not perceived to influence the care provided, emphasizing the delivery of the same or equal care: ***P003:**** “Every patient like doesn't matter, what kind of, if they are minority of culture, language or whatever, I just explained what I can do for them [the same] to everyone.”*

This dialogue highlighted differing opinions on culturally congruent care, with some embracing lived experiences and empathetic practices while others advocating for equal care.

Other culturally competent behaviours that participants highlighted included improving accessibility by offering intake forms in multiple languages, implementing sliding cost scales based on financial need, community outreach to diverse groups, and incorporating inclusive language practices (e.g., translation services).

### Understanding patients’ cultural values

It was evident that participants were aware that patient’s cultural values could influence their treatment, perceptions of health outcomes and how they describe their symptoms:

***P012:**** “I can only imagine that the way someone describes pain from a different culture will be different than the way I describe pain right, so I would say, you know we get a lot of like achy pain around here right now and, like you know what does achy mean you know what I mean. And someone from somewhere else will definitely say it differently.”*

However, it is worth noting that these distinctions in perception of symptoms were sometimes only tied to culture. Several participants acknowledged that variations in perception and expectations of care may not be attributed to cultural differences among equity-deserving groups:

***P010:**** “I wouldn't describe [pain perception] as being something that I would attach to any group, be that minority or whatever diversity. I mean there's absolutely variance in how individuals experience pain, but I don't apply that to any group, as you know, an expectation.”*

Participants described the importance of understanding patients' cultural values to address fears, mistrust of the healthcare system, and uncertainties related to treatment. They highlighted clinicians' responsibility to communicate and educate patients, especially when chiropractic care is unfamiliar, to provide empathetic, individualized care that respects diverse backgrounds.

## Contextual determinants of cultural competence in accessing care

This third and final theme, Contextual determinants of cultural competence in accessing care, captures causal factors related to health and illness external to the patients, providers, and clinical interaction. While these factors may not be culturally dependent, they could exacerbate the differences among equity-deserving groups in accessing chiropractic services. Such factors are included in subthemes 1) Patients’ Socioeconomic status, 2) Lack of Accessibility Limiting Access to Care, 3) Chiropractors as Advocates for Patient Equity.

### Patients’ socioeconomic status

Participants highlighted how socioeconomic status can significantly impact patients' ability to seek medical care and treatment effectiveness. They acknowledged the complex interplay between various socioeconomic factors and their intersection with culture, particularly in the context of equity-deserving groups. One participant *(P003)* described the connection between income and quality of life, explaining that individuals with higher incomes have better access to health care and a greater appreciation of its importance:

*P003: “Well, of course, quality of life depends on income, like the better work they have and usually gives them the ability to pay for more care, to pay for more good food, to have more free time, things like that, and they will have a better quality of life, overall. They will see the importance of getting care and taking care of their health, whereas people with lower income will ask, you know, if you don't eat, then paying for other health services is kind of not the first thing that comes to mind.”*

This quote highlights how financial disparities impact healthcare decisions. Other participants discussed the challenges of serving a community with a low socioeconomic profile, where many patients have limited education that affects their ability to navigate healthcare systems, communicate health concerns, and follow treatment plans. Participants noted that equity-deserving groups often face greater struggles between health needs and personal challenges:

***P014:**** “Over here we're dealing with a lot of individuals that don't even have a high school diploma…It causes a lot of disparities in healthcare, where accessing adequate healthcare services is difficult. Patients find it difficult to articulate their pain complaints and their concerns. Patients find it difficult to come for subsequent treatments in order to complete the treatment plan. So, there is a constant fight between healthcare, I would say, and personal finance, personal struggles.”*

Further, participants noted the importance of health literacy in providing care. One participant *(P003)* described how limited education among equity-deserving groups may influence their understanding of health care instructions: *“Their education is limited, so they kind of face that barrier as well, we have to make sure that we adapt to, the instruction has to be clear and easy to understand and easy to accomplish at home.”* Generally, participants highlighted the need for clear and effective communication to help bridge the gap patients experience because of a lack of health literacy.

### Lack of accessibility limiting access to care

Accessibility challenges significantly impact individuals from equity-deserving groups accessing chiropractic care. Participants noted that many clinics are situated in affluent areas, deterring patients with physical disabilities or lower socioeconomic status. One participant (P011) remarked, “*My clinic poses a lot of barriers to people right now*,” highlighting that their basement clinic lacks wheelchair access, making it difficult for those with disabilities, minorities or of lower income to seek care. Another participant echoed the significance of clinic facilities with adequate accessibility, emphasizing the need for wheelchair accessibility and even exploring options such as mobile care to reach patients who are unable to attend the clinic physically: ***P001:*** “*Making sure our clinic is, you know, wheelchair accessible is a big thing for like that kind of stuff. So, if we are treating those, those people, do they have access physically to our clinic, is a big thing, and then, are there ways that we can provide care outside of our treatment, like mobile, for example*.”

Nearly all participants acknowledged that the cost of treatment and lack of coverage within universal health plans disproportionately affect certain groups. One participant described many patients who rely heavily on insurance coverage, only deserving treatment if covered by their insurance, and often discontinuing care once their benefits are exhausted:

***P013:**** “A lot of people, then they rely on their insurance they won't come in, unless they've got insurance coverage and they only come into the extent that the insurance will provide […] and usually once the benefits are used up, they stop coming in, regardless of what their condition may be.”*

Participants also considered transportation a fundamental determinant impacting patients’ access to and utilization of care, as reflected by one participant (P011): “*A lot of people will say, well, I don't have access to the bus. […] That's also a factor of cost because they can't afford to take public transportation, and therefore, they end up you know not being able to show up for their appointment.”* Collectively, these barriers highlight systemic issues related to accessibility that impact access to chiropractic care for equity-deserving groups.

### Chiropractors as advocates for patient equity

Participants noted that individuals from equity-deserving groups often encounter challenges when navigating the healthcare system. One participant elaborated on this issue, describing how many patients often do not receive the care they need:

***P007:**** “Their experience in the Western medical model is a negative experience, and so when they come to me, and I change that experience for them, then they want to come to me for anything. [..] I had a patient who had obvious problems with weight loss and with bleeding where there shouldn't have been, and we had to really advocate for that patient to be referred down to cancer care and the medical doctor prior to that point had just said, ‘Oh, you're fine just take Tylenol.’ […] and thought they were like drug deserving or whatnot and labelled in a discriminatory way..”*

This participant highlighted cultural competency issues in the Canadian healthcare system for equity-deserving groups and the role of chiropractors in advocacy.

## Discussion

This study explores chiropractors’ experiences and perceptions in delivering care to equity-deserving groups in Canada and examines the factors that shape their ability to provide culturally competent care. We identified three main themes that reflect chiropractors’ experiences and perceptions of multilevel influences, both institutional and individual, that participants suggest could shape their ability to provide culturally competent care: perceived role of institutions to advance cultural competency, fostering a culturally responsive clinical practice, and understanding the contextual determinants in accessing care. Participants emphasized the role of institutions (e.g., associations, regulatory bodies, and educational organizations) in advancing DEI, addressing systemic racism, and increasing representation in leadership. At the clinician level, fostering culturally responsive clinical practice was integral to improving care for equity-deserving groups, as it related to cultural awareness and sensitivity, competency, and cultural values. Lastly, broader contextual factors, including socioeconomic status and lack of accessibility, highlight the opportunity for chiropractors to advocate for patients. These findings suggest a holistic approach to creating an inclusive chiropractic profession.

At the institutional level, participants highlighted the importance of more decisive action by academic institutions, regulatory bodies, and associations to address DEI. Participants highlighted the significant role of these institutions in advocating for dismantling systemic racism, reducing barriers to accessing care, improving education, and improving gender and cultural representation within leadership. This aligns with previous studies that identified gaps in chiropractic education regarding equity and cultural competency [15, 16] and the persistence of a lack of clinician diversity [15, 16] and gender disparities in leadership [35] across chiropractic and allied health professions. Additionally, current frameworks for delivering culturally congruent care highlight the need for systemic and clinician-level approaches [36]. Chiropractic institutions have increased efforts to address health inequities—through changes in policy at the Canadian Memorial Chiropractic College and DEI training offered through the Canadian Chiropractic Association. However, participants highlighted a more comprehensive, sustained approach involving engagement with key decision-makers (e.g., government) and equity-deserving groups. Previous research suggests that skills related to cultural competency cannot be taught during one educational session but rather through an ongoing educational journey with thoughtful reflection [37]. To meaningfully address these gaps, institutions could improve outreach programs to equity-deserving groups, engage with decision-makers, improve cultural and gender diversity within leadership positions, and consider embedding DEI within the curriculum. [38]

At the clinician level, fostering a culturally responsive clinical practice was considered essential to reducing disparities in the delivery of chiropractic care to equity-deserving groups. While previous research notes high cultural awareness and sensitivity among Canadian chiropractors [15], our results suggested inherent biases. All participants were able to identify culturally competent behaviours they engage in to enhance the delivery of care; however, there was variability in what they considered culturally competent behaviours. There appeared to be a comprehension gap among participants in our study when distinguishing between equitable and equal care and the implications for equity-deserving groups. Equitable care encompasses tailored approaches that acknowledge and address specific patient needs and barriers, aiming to mitigate existing health disparities [39–41]. In contrast, while well-intentioned, equal care might inadvertently overlook the systemic factors contributing to these disparities, potentially perpetuating unequal health outcomes [42]. This misunderstanding could undermine efforts to provide effective and unbiased healthcare solutions for equity-deserving populations. A healthcare provider deemed culturally competent is an individual who can interact appropriately with other people—by acknowledging and addressing systemic barriers that may lead to health disparities [43]. High cultural competency can improve clinicians’ knowledge, attitudes, and skills [44], improving empathy for patients from diverse cultural and socioeconomic backgrounds [45]. There is a need to develop targeted, ongoing training to enhance cultural competency among chiropractors.

Participants also identified contextual factors that may influence the delivery of chiropractic care to equity-deserving groups. Specifically, there is a lack of accessibility related to the clinics’ structure for people with disabilities and high treatment costs, as noted in previous literature [15]. Additionally, participants highlighted lower socioeconomic status and lack of health literacy may limit patient accessibility to chiropractic services. It is important to consider how the intersectionality of social factors (e.g., sex and lower income, race/ethnicity and lower income, among others) may disproportionally affect equity-deserving groups, leading to disparities in health outcomes [1–7]. Understanding these overlapping social factors is essential to addressing health disparities, as the cumulative effect of these challenges can contribute to poor health outcomes and reduced quality of life for affected individuals. Institutions and clinicians should address contextual barriers by adopting more inclusive practices and fostering physically and culturally accommodating environments for diverse groups.

## Implications

Culturally congruent care involves top-down and bottom-up approaches integrating DEI practices at institutional and clinician levels.^36^ Institutions should prioritize incorporating DEI training within curricula, create policies that foster diversity, and advocate for accessibility initiatives to reduce treatment costs. Clinicians should engage with equity-deserving groups to understand unique cultural needs and tailor treatments to each patient rather than a one-size-fits-all. Integrating intersectionality training ensures that clinicians understand how overlapping social identities can compound health disparities. Regular evaluation of DEI initiatives through patient feedback and outcome measures may help the profession become more accessible and responsive to the needs of all Canadians.

## Strengths/limitations

This study included a convenient sample of chiropractors who were likely informed and agreed with DEI concepts; we did not capture individuals who disagreed with these concepts. Future studies should include participants who disagree with DEI initiatives to understand those perspectives better and identify barriers to acceptance, resistance factors, and potential strategies for fostering inclusive dialogue within the profession. Social desirability bias may have played a role in participants' responses. Additionally, we did not include the patient perspective in this study, so it is uncertain if the chiropractor’s perspective is congruent with their patients. We asked participants about their experiences with equity-deserving groups based on cultural differences (e.g., race, sex, gender, etc.); however, it is unclear how participants perceived this definition. Future studies should address specific equity-deserving groups and their barriers to accessing care. The strength of this study is that minimal information exists within the chiropractic profession regarding this topic. This study provides novel information about DEI within the profession.

## Conclusion

The results highlight several important considerations and strategies for providing chiropractic care to equity-deserving groups in Canada. These findings may inform the development of profession-specific training in cultural competence and policy development. Future research should aim to understand the provision of culturally competent care within the chiropractic profession.

## Data Availability

The data that support the findings of this study consist of qualitative interviews and transcripts, which are not publicly available due to privacy and confidentiality concerns. Participants consented to the use of their data for research purposes under the assurance that their identities and personal information would remain protected. As such, sharing raw data publicly is not feasible.Researchers interested in accessing anonymized excerpts or further information about the data are encouraged to contact the corresponding author. Access to data will be considered on a case-by-case basis, in accordance with ethical guidelines and data-sharing agreements, and may require additional institutional review board approval.

## Appendix A: Guide for qualitative interviews

Thank you for agreeing to participate in this study. The goal of this interview is to understand cultural health disparities as they relate to the provision of Canadian rehabilitation services. We are trying to get a sense of the challenges and successes you’ve had in treating people from a minority community and the potential changes that can be made to better serve them. A minority community, in the context of this interview, will be defined as differences among groups of people and individuals based on ethnicity, race, socioeconomic status, gender, exceptionalities, language, religion, sexual orientation, and geographical area. This interview will be audio recoded, are you okay with this? I may also be taking notes during our conversation. Do you have any questions for me before we get started?

Tell me a little bit about your clinical practice and the community in which you practice.

- How would you describe the demographic make-up of your clinic, in terms of the patients that you see?

What has been your experience with different minority groups accessing physiotherapy?

- Have you experienced any differences regarding health outcomes of minority communities as compared to other populations? (For example, differences in pain management, quality of life, satisfaction with care, etc.)
  - If yes, can you share an example?
  - Why do you think these exist?
- How do you approach the delivery of your health care services to your patients? Does belonging to a different cultural group play a role in the type of care provided? How? Why?
- How do you modify your treatment interventions to accommodate different cultural practices? (e.g., gowns available to patient that are uncomfortable)
  - Do you think you have sufficient cultural knowledge to provide treatment?
- From your perspective, do you think that diverse communities are perceiving chiropractic or physiotherapy care differently?
  - E.g., individuals that come from different cultural where chiropractic/physiotherapy is not utilized may have different ideology of what to expect
- How do you use your cultural knowledge of diverse communities to modify your treatment interventions?
  - How do you perceive your level of cultural knowledge to provide treatment? Probe: do feel this is sufficient or adequate?
- How do you think patients from diverse backgrounds define/experience pain and/or disability?
- What do you think are the main factors that influence delivery of chiro or physio services to patients from minority communities?
  - Probe: Language, cost, communication, education, time per treatment
- Do you believe that personal clinician biases (subconscious or conscious) play a role in influencing the delivery of care?
- In your opinion, what can health care providers do to help eliminate health care disparities as they relate to minority communities?

Do you have any office procedures/protocols around the policy of providing equitable patient care?

- If yes, what?
- If no, is there anything you would consider implementing? What resources would you require to provide equitable care?
- Are these policies written? Implied?

In your opinion, how can clinical practice be tailored to meet the needs of diverse communities?

Probe: consent forms in multiple languages, easily accessible translational services, etc.)

In most of the health care professions the majority of providers are Caucasian (white). Do you think this impacts health care practice? What steps can be taken to increase diversity, in clinical practice?

Is there anything else you would like to tell me about?
