# Correction: Factors that influence the delivery of chiropractic services to equity-deserving groups in Canada: a qualitative study

**DOI:** 10.1186/s12998-025-00623-x

**Published:** 2025-11-27

**Authors:** Nora Bakaa, Stephanie DiPelino, Danielle Southerst, Silvano Mior, Lisa Carlesso, Joy MacDermid, Luciana Macedo

**Affiliations:** 1 Department of Rehabilitation Sciences, McMaster University, 1280 Main St. W, Hamilton, ON Canada; 2 Institute for Disability and Rehabilitation Research, Ontario Tech University, Oshawa, ON Canada; 3 Department of Research and Innovation, Canadian Memorial Chiropractic College, Toronto, ON Canada; 4 School of Physical Therapy, Western University, London, ON Canada

**Correction to: Chiropractic & Manual Therapies (2025) 33:18** 10.1186/s12998-025-00582-3

The original publication of this article contained an incorrect definition in the introduction paragraph. The incorrect and correct information is listed in this correction article. The original article has been updated.

Incorrect

While equity-deserving groups actively advocate for systemic change to address barriers to healthcare access, equity-seeking groups inherently warrant equitable care based on social justice and inclusion principles [8]. These terms are often used interchangeably, but their distinction is crucial: equity-deserving reflects activism for change, whereas equity-seeking emphasizes the fundamental right to healthcare without requiring advocacy.

Correct

While equity-seeking groups actively advocate for systemic change to address barriers to healthcare access, equity-deserving groups inherently warrant equitable care based on social justice and inclusion principles [8]. These terms are often used interchangeably, but their distinction is crucial: equity-seeking reflects activism for change, whereas equity-deserving emphasizes the fundamental right to healthcare without requiring advocacy.

